# Phylogenetic Analysis of *Giardia lamblia* Human Genotypes in Fars Province, Southern Iran

**Published:** 2017

**Authors:** Mohammad RAYANI, Gholamreza HATAM, Ngah Zasmy UNYAH, Abdolmajid ASHRAFMANSORI, Wan Omar ABDULLAH, Rukman Awang HAMAT

**Affiliations:** 1. Dept. of Microbiology and Parasitology, School of Health, Bushehr University of Medical Sciences, Bushehr, Iran; 2. The Persian Gulf Tropical Medicine Research Center, Bushehr University of Medical Sciences, Bushehr, Iran; 3. Dept. of Medical Microbiology and Parasitology, Faculty of Medicine and Health Sciences, University Putra Malaysia, Serdang, Malaysia; 4. Basic Sciences in Infectious Diseases Research Center, School of Advanced Medical Sciences and Technologies, Shiraz University of Medical Sciences, Shiraz, Iran

**Keywords:** *Giardia lamblia*, Genetic variation, Glutamate dehydrogenase (*gdh*), Iran

## Abstract

**Background:**

This study is the first phylogenetic genotype analysis of *Giardia lamblia* in Iran. The main objective was to determine genotyping and identify the sub-assemblages of *Giardia lamblia* isolates involved in the transmission of giardiasis in Fars Province, south of Iran, in 2012.

**Methods:**

Forty *G. lamblia* isolates were collected from the patient’s fecal samples with gastrointestinal discomfort referred to the health centers and hospitals in Shiraz, Fars Province, south of Iran. Purification of *G. lamblia* cysts from fecal samples and DNA extraction were performed using monolayer of sucrose density gradient and Phenol-Chloroform-Isoamylalcohol (PCI) respectively. Semi-nested PCR and sequence analysis were then performed using the primers (GDHeF, GDHiF, and GDHiR) which amplified a 432-bp fragment of *Giardia* glutamate dehydrogenase (*gdh*) gene. Phylogenetic analysis was carried out using a neighbor-joining tree composed of the nucleotide sequences of *G. lamblia* isolates obtained in this study and the known sequences isolates published in GenBank.

**Results:**

*G. lamblia* sub-assemblage AII was the most prevalent genotype with 80% of the cases and 20% of the cases belong to sub-assemblage BIII and BIV based on the DNA sequence of the *gdh*. *G. lamblia* isolates at Fars Province were widely distributed within assemblage A cluster (sub-assemblage AII) and the remaining isolates were dispersed throughout the assemblage B cluster (sub-assemblage BIII and BIV).

**Conclusion:**

PCR Sequencing and phylogenetic analysis was a proper molecular method for genotyping and discriminating of the of *G. lamblia* sub-assemblages in fecal samples, using the glutamate dehydrogenase gene that suggests a human contamination origin of giardiasis.

## Introduction

The taxonomy of *Giardia* has revealed six *Giardia* species: *G. lamblia* in mammals, including human; *G. agilis* in amphibians; *G. ardeae* and *G. psittaci* in birds; *G. microti* and *G. muris* in rodents (1). *G. lamblia* (synonym of *G. intestinalis* and *G. duodenalis*) is the most common intestinal protozoan parasite of a wide range of mammalian hosts and has a global distribution (2, 3). *G. lamblia* is a species complex with eight major genotypes or assemblages (A–H). Molecular characterizations based on multiple loci are used as the main tool to study genetic variation of different populations of *Giardia* (4, 5). Phylogenetic analysis using the small subunit ribosomal RNA (SSU-rDNA), glutamate dehydrogenase (*gdh*), beta-giardin (*bg*) and triose phosphate isomerase (*tpi*) genes have been used to study genetic variability and relations within assemblages of *G. lamblia* from different hosts. The *gdh* locus has been used to successfully genotype isolates of *G. lamblia* from a range of vertebrate hosts and it allows the discrimination between the various subgenotypes of *G. lamblia* assemblages (6, 7). In this study, glutamate dehydrogenase marker was used in the molecular characterization methods for genotyping and subtyping the isolates of *G. lamblia* from patients.

*G. lamblia* is one of the most common intestinal parasites in Iran. The prevalence of giardiasis varies in different parts of Iran (5%–23%) (8). Molecular epidemiology of human giardiasis is still unclear in Iran. Previously, there was no study performed using phylogenetic technique for *G. lamblia* in Iran. Only little information was available on the *G. lamblia* genotypes. Three studies based on PCR-RFLP assay, targeting the *gdh* gene of *G. lamblia* isolates genotype in Tehran, Shiraz and Isfahan provinces showed that assemblage A was the most prevalent (9–11). In contrast, two studies based on PCR-RFLP assay, targeting the *gdh* gene of *G. lamblia* isolates in Tabriz and in Uremia showed that assemblage B was the most prevalent genotype (12, 13).

Molecular studies on *G. duodenali* isolates and discrimination of genotypes is a useful way to know the transmission route and effective prevention management of giardiasis. The objective of this study was to determine genotyping and identify the sub-assemblages of *G. lamblia* isolates involved in the transmission of giardiasis in Fars Province, south of Iran. Genotypic characterization of the *gdh* gene was performed by using genomic DNA directly extracted from human fecal samples to evaluate the potential transmission routes of *G. lamblia* in this area.

## Materials and Methods

### Sample collection

Forty *G. lamblia* isolates were collected from the patient’s fecal samples with gastrointestinal discomfort referred to the health centers and hospitals in Shiraz, Fars Province, South of Iran in 2012. Then, the samples were sent to the Research Laboratory of Intestinal Protozoa in the Department of Parasitology and Mycology in School of Medicine at Shiraz University of Medical Sciences for further examination. Informed consent was taken from the patients and the study was approved by Ethics Committee of the university.

### Purification technique

Purification of *G. lamblia* cysts, which were to be used for DNA extraction, was done using the monolayer sucrose density gradient technique. Single step sucrose gradient with the specific gravity at 0.85 M was performed on positive stool samples in order to concentrate the cysts from fecal samples (14). Aliquots of the purified fecal samples were stored at 4 °C and −20 °C.

### DNA extraction

The genomic DNA of *G. lamblia* was extracted using the Phenol-Chloroform-Isoamylalcohol (PCI) method with pretreatment by Triton X100 on purified fecal samples (14). DNA extracts were stored at −20 °C for PCR analysis.

### PCR amplification of gdh gene

A semi-nested PCR was performed using three primers known as GDHeF, GDHiF, and GDHiR that amplify a 432-bp fragment of the *gdh* gene (7) with some slight modifications. PCR reaction mixtures consisted of 12.5 pmol of each primer, 200 μmol of each dNTP, 1.5 mM of MgCl_2_, were carried out in 25 μl volumes on a Corbett Research Thermal Cycler, Australia with the following amplification conditions: one cycle of 94 °C for 2 min, 56 °C for 1 min and 72 °C for 2 min, followed by 31 cycles of 94 °C for 30 sec, 56 °C for 20 sec and 72 °C for 45 sec and a final extension of 72 °C for 7 min. Both positive and negative controls were included in each round of PCR to validate results. One microliter of PCR product from the primary reaction was added to the secondary PCR containing primers GDHiF and GDHiR. Reactions were visualized in UV on 1.5% agarose gels stained with ethidium bromide.

### Sequence analysis of gdh gene

Forty of the PCR products successfully amplified for the *gdh* locus were sent to First BASE Laboratories (http://www.base-asia.com) for commercial DNA sequencing reactions in both forward and reverse directions using primers GDHiF and GDHiR. Nucleotide sequences were analyzed using the computer program of sequencing scanner software. Multiple alignments and sequence alignment of these sequences were carried out using the BioEdit and the ClustalW MEGA4 programs. The *gdh* sequences of *G. lamblia* obtained in this study were aligned with previously published reference sequences of *G. lamblia* isolates from the GenBank database (Table 1). DNA sequencing of PCR products compared with already known *G. lamblia* sequences obtained from GenBank using ClustalW in the MEGA4. The accession numbers obtained for each reference assemblages from GenBank were L40509 (AI), L40510 (AII), AF069059 (BIII), L40508 (BIV), U60984 (C), U60986 (D), U47632 (E), AF069057 (F) and AF069058 (G) (5, 6, and 15). To determine similarities or homologies to known genes in NCBI GenBank sequence database, all obtained nucleotide sequences were submitted to BLAST searches. Sequence representatives for each of the assemblages identified in the studies were submitted to GenBank.

**Table 1:** *Giardia gdh* gene sequences available in GenBank used in this study

***Isolate***	***GenBank accession number***	***Sub-assemblage***	***Country***	***Reference***
Ad-1	L40509	AI	Australia	(6)
Portland 1	M84604	AI	USA	(16)
Ad-1	AY178735	AI	Australia	Unpublished data
F22	EF507606	AI	Brazil	(17)
Ad-2	L40510	AII	Australia	(6)
Ad-113	AY178736	AII	Australia	Unpublished data
H32	EF507674	AII	Brazil	(8)
NLH20	AY826194	AII	Netherland	(5)
GH-125	AB195222	AII	Japan	(18)
TIG20	AB434776	AII	Iran	Unpublished data
T11	JF968202	AII	Iran	Unpublished data
16	JF917086	AII	Iran	Unpublished data
Bah-12	AF069059	BIII	Australia	(19)
FCQ-21	AY178756	BIII	Australia	Unpublished data
Cub-G89	EU594665	BIII	Cuba	(20)
Cub-G81	EU594667	BIII	Cuba	(20)
gd-ber9	DQ090540	BIII	Norway	(21)
gd-ber10	DQ090541	BIII	Norway	(21)
TIG12	AB434535	BIII	Iran	Unpublished data
A14	JF968198	BIII	Iran	Unpublished data
Ad-7	L40508	BIV	Australia	(6)
Ad-28	AY178738	BIV	Australia	Unpublished data
Ad-45	AY178739	BIV	Australia	Unpublished data
Ad-85	AY178755	BIV	Australia	Unpublished data
NLH13	AY826191	BIV	Netherland	(4)
H30	EF507672	BIV	Brazil	(17)
GH-156	AB182126	BIV	Japan	(22)
GH-158	AB188825	BIV	Japan	(23)
Ad-141	U60984	C	Australia	(15)
Ad-148	U60986	D	Australia	(15)
P-15	U47632	E	Australia	(24)
Ad-23	AF069057	F	Australia	(19)
Ad-157	AF069058	G	Australia	(19)
*G. ardeae*	AF069060		Australia	(19)

### Phylogenetic analysis of gdh gene

Phylogenetic analysis was performed using Neighbor-joining (NJ) method in the MEGA4 program. The evolutionary distance-based analysis was conducted using the Kimura 2-parameters model to estimates the distance among assemblages identified in this study. For each calculation, branch reliability was assessed using bootstrap analysis (1000 replicates). A neighbor-joining (NJ) tree composed of the 40-nucleotide sequences of *G. lamblia* isolates obtained in this study and 33 sequences of *G. lamblia* isolates of the *gdh* gene in the GenBank database (Table 1) was reconstructed. The homologous nucleotide sequence of *G. ardeae* (GenBank accession number AF069060) was used as outgroup.

## Results

### PCR amplification of gdh gene

**Fig. 1:**
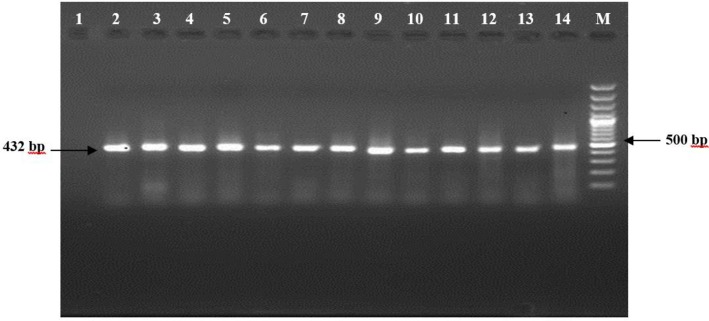
Electrophoresis of PCR products with *gdh* primers (expected size of 432 bp) Lane 1 = Negative Control (primers + buffer) // Lane 2 = Positive control Lane 3–14 = *G. lamblia* isolates from patients // Lane M = 100 bp DNA marker (Fermentas, Canada)

The *Giardia gdh* locus with 432 bp was successfully amplified by semi-nested PCR. Fig. 1 shows a representative gel electrophoresis photograph of the PCR products amplified with *gdh* primers.

### Sequence analysis of the gdh gene

Sequence analysis was performed on 40 PCR products of *G. lamblia* based on *gdh* amplification both directions using GDHeF, GDHiF and GDHiR primers.

**Fig. 2:**
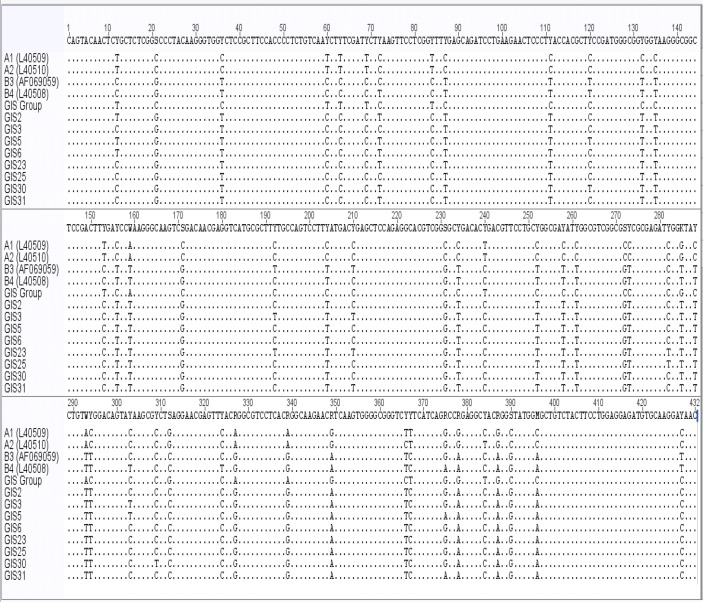
Sequence analysis for *gdh* gene nucleotide substitutions of *G. lamblia* isolates obtained from this study compared with reference sequences

The genotyping results indicated the presence two main genotypes A and B of *G. lamblia*. Based on the DNAsequences of the glutamate dehydrogenase, 32 isolates (80%) were detected as assemblage A and 8 isolates (20%) were identical to assemblage B. After performing a BLAST search, analysis revealed all 32 isolates assemblage A sequences were 100% similar to sub-assemblage AII (GenBank Accession number L40510). From 8 isolates as assembalge B, three isolates were 99% similar to sub-assemblage BIII (GenBank Accession number AF069059), one isolate was 99% similar to sub-assemblage BIV (GenBank Accession number L40508) and four isolates were found between sub-assemblage BIII and BIV (Table 2). Comparison of *gdh* gene nucleotide substitutions between *G. lamblia* isolates obtained from this study and GenBank database published reference has shown in Fig. 2.

**Table 2:** Genotyping characterization of 40 *G. lamblia* patients isolates from Fars Province, Iran

***Patient isolates***	***Sub-assemblages***
32 *G. lamblia* isolates (GIS Group) (80%):	AII (L40510)
GIS1, GIS4, GIS7, GIS8, GIS9, GIS10, GIS11, GIS12, GIS13, GIS14, GIS15, GIS16, GIS17, GIS18, GIS19, GIS20, GIS21, GIS22, GIS24, GIS26, GIS27, GIS28, GIS29, GIS32, GIS33, GIS34, GIS35, GIS36, GIS37, GIS38, GIS39 and GIS40	
3 *G. lamblia* isolates (7.5%):	BIII (AF069059)
GIS3, GIS23 and GIS25	
1 *G. lamblia* isolates (2.5%):	BIV (L40508)
GIS5	
4 *G. lamblia* isolates (10%):	Between BIII & BIV
GIS30, GIS31, GIS2 and GIS6	

Accession numbers for reference sequences obtained from GenBank are given in parentheses

### Phylogenetic analysis of gdh gene

**Fig. 3:**
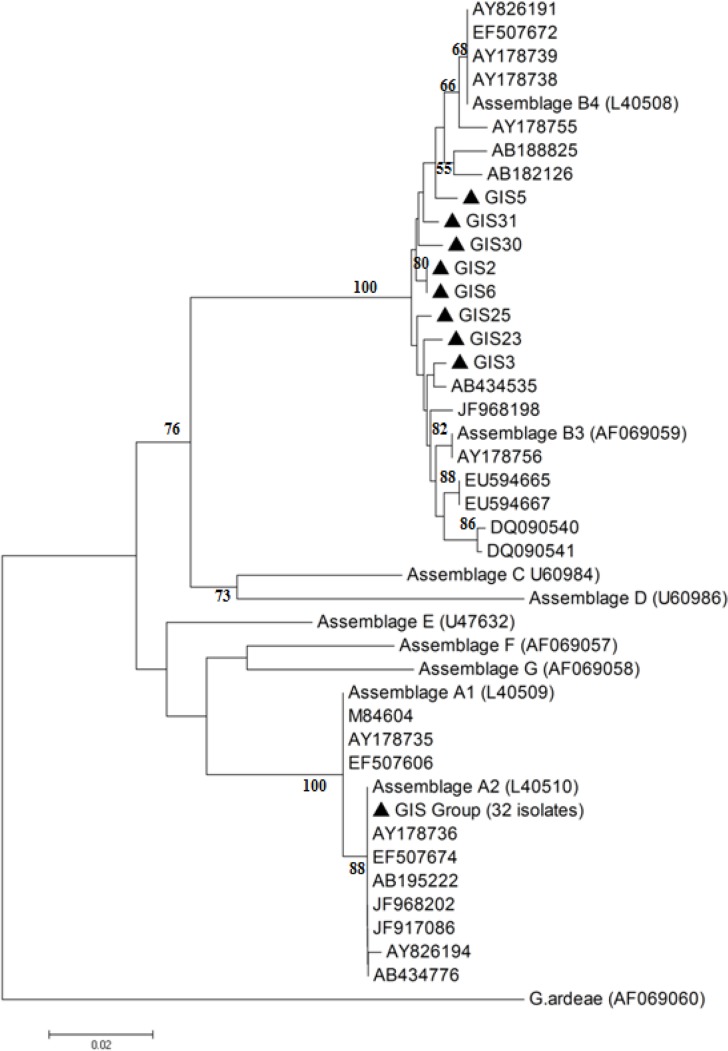
Phylogenetic relationships of *G. lamblia* isolates in this study and reference isolates and other *G. lamblia* assemblages previously published in GenBank (Table 1) inferred by Neighbor-Joining analysis of Kimura’s distances calculated based on the nucleotide sequences of *gdh* with the MEGA4 program. Bootstrap values #50% are not shown. Accession numbers for reference sequences obtained from GenBank are given in parentheses. The sequence of *Giardia ardeae* was used as an out-group ▲ is the indicator for Iranian isolates.

Phylogenetic analysis of *gdh* DNA sequences was determined to further clarify the relationship of the different genotypes to each other. Fig. 3 shows the Phylogenetic tree based construction on the neighbor-joining method of the *gdh* sequences from *G. lamblia* of patients isolates determined in this study and GenBank reference isolates and other *G. lamblia* assemblages previously published (Table 1).

The phylogenetic tree shows that assemblage A was the most frequent, corresponding with 32 cases (80%). All isolates in cluster A were distributed in sub-assemblage AII. The second cluster was contained the human assemblage B isolates. Assemblage B was found in 8 cases (20%) of the studied samples. Resulting in assemblage B revealed 2 major lineages designated as assemblages BIII and BIV. Third and fourth were the dog clusters of either assemblage C or D. The fifth cluster was contained the cattle assemblage E. The sixth was contained the cat assemblage F cluster. The seventh was contained the rodent assemblage G cluster. Finally, the eighth cluster was contained the *G. ardeae* (Fig. 3). Phylogenetic analysis of the *gdh* gene of *G. lamblia* isolates from patients provided strong bootstrap support (100%) for the placement of genotypes A and B in separate clusters, indicating the differentiation of the assemblages A and B. Phylogenetic analysis has shown Fars isolates were widely distributed within assemblage A cluster (sub-assemblage AII) and the remaining Fars isolates were dispersed throughout the assemblage B cluster (sub-assemblage BIII and BIV). No samples were joined in clusters corresponding to assemblages C, D, E and F and G of *G. lamblia*, also in sub-assemblage AI.

## Discussion

Recent progressions on the identification of *Giardia* assemblages and sub-assemblages have increased tremendously in our understanding of the biology and host-parasite relationship of *G. lamblia*. In this study, 40 known *G. lamblia* isolates from patients were used to determine the sub-assemblages that occur in Fars province, Iran. All 40 *G. lamblia* isolates can be divided into four main groups based on the results obtained from this study. These groups are (i) sub-assemblage AII, (ii) sub-assemblage BIII, (iii) sub-assemblage BIV and (iv) sub-assemblage BIII/BIV as shown in Table 2. The sequences of *gdh* for the entire 40 *G. lamblia* patient isolates were compared to the sequences of known *G. lamblia* assemblages published by GenBank (Table 1).

In our study, three *G. lamblia* patient isolates were associated with sub-assemblage BIII and one isolate was associated with sub-assemblage BIV. Four isolates was situated between sub-assemblage BIII and BIV (Table 2 and Fig. 3). Additionally, Sequencing and phylogenetic analysis of *gdh* gene sequences from this study and a large set of those which were available from the literature worldwide showed the sequences of the 32 isolates associated with sub-assemblage AII were highly genetically homogenous with 100% homology to published sequences.

While the other eight isolates associated with sub-assemblages, BIII and BIV showed variability within the study area.

The results of this study are similar to previous studies (9–11) in which *G. lamblia* cysts isolated from human feces were analyzed with PCR-restriction fragment length polymorphism (RFLP) assay, based on the detection of glutamate dehydrogenase (*gdh*) genes. In Tehran, the capital of Iran a majority of 87% of the *G. lamblia* isolates used in the studies were from sub assemblage AII, whereas 7.8% of the isolates were from sub-assemblage BIII (9). Moreover, In Fars Province, 74.41% isolates were typed as assemblage AII, 17.44% as assemblage BIII, 3.49% as assemblage BIV and 4.66% isolates as mixed assemblages AII and BIV (10). In Isfahan Province in the center of Iran, PCRRFLP, genotype A group II was detected in 59.7% isolates compared to genotype B that showed 34.32% samples as genotype B Group III and 2.98% sample as genotype B group IV (11). In contrast with our study, assemblage B was predominant in Tabriz and Uremia cities in the northwest of Iran when the *gdh* gene was targeted by PCR RFLP. In Tabriz analysis, PCR-RFLP revealed that 28 samples (33.3%) were in sub-assemblage AII and 44.4% and 22.2% belonged to sub-assemblages BIII and BIV, respectively (12). In Uremia, 93.3% of isolates were found to be related to the genotype BIII and 6.7% were related to the subgroup BIV (13).

Current study had focused on the genetic characterization of *G. lamblia* at the *gdh* gene using DNA samples from patients’ stool samples in Fars Province, Iran. Previous surveys performed on DNA extracted directly from fecal samples in various countries have confirmed that only *G. lamblia* assemblages A and B are associated with human infections (25). Genotypic analysis showed that only combination of assemblages AII and B are able to infect humans in this area of Iran, suggesting a human contamination origin. Most of our patients with sub-assemblage AII infection had acquired their parasites through the anthroponotic route, mainly involved human-to-human transmission. Zoonotic transmission may also occur. Reservoir sources of zoonotic transmission were found to be a number of livestock animal species like dairy cattle, companion animals like cats, dogs and wildlife animals (26). Unfortunately, due to limited funding, no animal sample was included in this study, so current result was not enough to demonstrate the role of anthroponotic and zoonotic aspects of giardiasis infections. Further molecular characterization of species, genotypes and subtyping analysis of specimens from both humans and animals and comparisons of various genetic loci are required for identification of the transmission of giardiasis between animals and humans and, understanding of giardiasis transmission.

Phylogenetic analysis of *G. lamblia* isolates from this study and previously published *G. lamblia* assemblages revealed two different clusters A and B. In cluster A there were two subclusters AI and AII. Within AI subcluster, three *G. lamblia* isolates Portland 1 (GenBank accession number: M84604-USA), F22 (EF507606-Brazil), and Ad-1 (AY178735-Australia) matched with reference isolate Ad-1(L40509-Australia). We did not have any sample of this study in this subcluster. Within subcluster AII, 32 homolog isolates of this study completely matched (GIS Group) with reference isolate Ad-2 (GenBank accession number: L40510-Australia). Moreover, other isolates Ad-113 (AY178736-Australia), TIG20 (AB434776-Iran), T11 (JF968202-Iran), 16 (JF917086-Iran), H32 (EF507674-Brazil), NLH20 (AY826194-Netherland) and GH-125 (AB195222-Japan) were in this subcluster. In cluster B there were two sub-clusters BIII and BIV with some subgroups that reveal variation in this assemblage. Within the BIII subcluster, reference isolate Bah12 (GenBank accession number: AF069059-Australia) and Fcq-21 (AY178756-Australia) matched in a subgroup. Isolates cub-G89 (EU594665-Cuba), cub-G81 (EU594667-Cuba), isolates gd-ber9 (DQ090540-Norway), gd-ber10 (DQ090541-Norway), and A14 (JF968198-Iran) did not match with Bah12. Moreover, the isolate GIS3 (this study) and TIG12 (AB434535-Iran) (in a subgroup) and 2 other isolates from this study GIS23 and GIS25 had variant with Bah12. In BIV sub cluster, Ad-28 (GenBank accession number: AY178738-Australia), Ad-45 (AY178739-Australia), H30 (EF507672-Brazil) and NLH13 (AY826191-Netherland) matched with reference isolate Ad7 (L40508-Australia). Isolate Ad-85 (AY178755-Australia) was found very close to this subgroup. Another two samples GH-158 (AB188825-Japan) and GH-156 (AB182126-Japan) (in a subgroup) and 5 of our samples GIS5, GIS31, GIS30 and GIS2 and GIS6 (in a subgroup), set far distance from reference isolate Ad7 in this subcluster. Analysis of the cluster B indicated genetic heterogeneity in assemblage B. On the other hand, separate samples from Japan, Cuba, Norway, and Iran having different sub category may suggest that in a country and in different countries in the world have different strains. Some isolates from this study (GIS23, GIS25, GIS2, GIS6, GIS30 and GIS31) that were very far distanced from the reference isolates in cluster B could be the new strains that need more investigations in future studies (Table 1 and Fig. 3).

Geographic distribution of each *Giardia* assemblage in humans varies in different parts of the world. Our results with predominance of assemblage A was same to studies from Brazil (27), Colombia (28), France (29), Iran (9), Italy (30), Mexico (31–33), New Zealand (34), Portugal (3), Thailand (35), United States (36) and Yemen (37). Some studies from Argentina (38), Australia (39, 40), Bangladesh (41), Belgium (42), Colombia (43), Egypt (44, 45), India (46), Malaysia (47), Morocco (48), Netherland (4), Nicaragua (49), Norway (23, 50), The Philippines (51), Thailand (52), United Kingdom (2, 53) and Sweden (54) with a predominance of assemblage B, differed from our study. Predominant *G. lamblia* sub-assemblage AII has also been shown in Australia (6), Bangladesh (41), Belgium (42), England (2), France (55), Iran (13), Mexico (33), Nicaragua (49), Peru (56) and The Philippines (51).

## Conclusion

Human giardiasis is one of the most common intestinal parasitic diseases in Iran, and its molecular epidemiology needs more investigations. PCR sequencing and phylogenetic analysis using the glutamate dehydrogenizes gene are proper molecular methods for *G. lamblia* genotyping and could discriminate *G. lamblia* sub-assemblages in fecal samples. The high prevalence of sub-assemblage AII in this study and other similar investigations conducted in Iran reemphasized the importance of applying all the health recommendations. Health education and improvement of sanitation conditions during drinking water and food supply are the most important basic strategies for the control and prevention of anthroponotic transmission of giardiasis in this community. The results could use in the future studies for clinical management and prevention policies.

## References

[1] Feng Y Xiao L Zoonotic potential and molecular epidemiology of *Giardia* species and giardiasis. Clin Microbiol Rev. 2011;24(1):110–40. 21233509 10.1128/CMR.00033-10 PMC3021202

[2] Amar CF Dear PH Pedraza-Díaz S Sensitive PCR-restriction fragment length polymorphism assay for detection and genotyping of *Giardia lamblia* in human feces. J Clin Microbiol. 2002;40(2):446–52. 11825955 10.1128/JCM.40.2.446-452.2002 PMC153413

[3] Sousa MC Morais JB Machado JE Genotyping of *Giardia lamblia* Human Isolates from Portugal by PCR RFLP and Sequencing. J Eukaryot Microbiol. 2006;53 Suppl 1:S174–6. 17169050 10.1111/j.1550-7408.2006.00221.x

[4] van der Giessen JW de Vries A Roos M Genotyping of *Giardia* in Dutch patients and animals: a phylogenetic analysis of human and animal isolates. Int J Parasitol. 2006;36(7):849–58. 16701663 10.1016/j.ijpara.2006.03.001

[5] Heyworth MF *Giardia lamblia* genetic assemblages and hosts. Parasite. 2016;23:13. 26984116 10.1051/parasite/2016013 PMC4794627

[6] Monis PT Mayrhofer G Andrews RH Molecular genetic analysis of *Giardia intestinalis* isolates at the glutamate dehydrogenase locus. Parasitology. 1996;112 (Pt 1):1–12. 8587793 10.1017/s0031182000065021

[7] Read CM Monis PT Thompson RC Discrimination of all genotypes of *Giardia lamblia* at the glutamate dehydrogenase locus using PCR-RFLP. Infect Genet Evol. 2004; 4(2):125–30. 15157630 10.1016/j.meegid.2004.02.001

[8] Taherkhani H Shariati S Abdolahi N Clinical manifestations of Giardiasis in Iran. Journal of Clinical and Diagnostic Research. 2009;3(2):1416–18.

[9] Babaei Z Oormazdi H Akhlaghi L Molecular characterization of the Iranian isolates of *Giardia lamblia*: application of the glutamate dehydrogenase gene. Iran J Public Health. 2008;37(2):75–82.

[10] Sarkari B Ashrafmansori A Hatam GR Genotyping of *Giardia lamblia* isolates from human in southern Iran. Trop Biomed. 2012;29(3):366–71. 23018499

[11] Pestehchian N Rasekh H Babaei Z Identification of genotypes of *Giardia lamblia* human isolates in Isfahan, Iran, using polymerase chain reaction–Restriction Fragment Length polymorphism. Adv Biomed Res. 2012;1:84 23946932 10.4103/2277-9175.105166 PMC3724326

[12] Fallah E Nahavandi KH Jamali R Molecular identification of *Giardia lamblia* isolates from human and animal reservoirs by PCRRFLP. J Biol Sci. 2008; 8(5):896–901.

[13] Hazrati Tappeh K Manafi G Asgharzadeh M Incidence of *Giardia lamblia* Subspecies by PCR-RFLP in Stool Specimens of Hospitalized Children at Urmia Mutahhari Hospital, West Azerbaijan Province, Iran. Iran J Parasitol. 2014;9(4):541–7. 25759735 PMC4345093

[14] Rayani M Zasmy Unyah N Hatam G Molecular Identification of *Giardia lamblia* Isolates from Fars Province, Iran. Iran J Parasitol. 2014;9(1):70–78. 25642262 PMC4289883

[15] Monis PT Andrews RH Mayrhofer G Novel lineages of *Giardia intestinalis* identified by genetic analysis of organisms isolated from dogs in Australia. Parasitology. 1998;116 (Pt 1):7–19. 9481769 10.1017/s0031182097002011

[16] Yee J Dennis PP Isolation and characterization of a NADP-dependent glutamate dehydrogenase gene from the primitive eucaryote *Giardia lamblia*. J Biol Chem. 1992; 267(11):7539–44. 1559991

[17] Souza SL Gennari SM Richtzenhain LJ Molecular identification of *Giardia lamblia* isolates from humans, dogs, cats and cattle from the state of Sao Paulo, Brazil, by sequence analysis of fragments of glutamate dehydrogenase (*gdh*) coding gene. Vet Parasitol. 2007;149(3–4):258–64. 17900812 10.1016/j.vetpar.2007.08.019

[18] Abe N Kimata I Tokoro M Genotyping of *Giardia* isolates from humans in Japan using the small subunit ribosomal RNA and glutamate dehydrogenase gene sequences. Jpn J Infect Dis. 2005;58(1):57–8. 15728998

[19] Monis PT Andrews RH Mayrhofer G Molecular systematics of the parasitic protozoan *Giardia intestinalis*. Mol Biol Evol. 1999;16(9):1135–44.. 10486969 10.1093/oxfordjournals.molbev.a026204

[20] Pelayo L Nuñez FA Rojas L *Giardia* infections in Cuban children: genotypes circulating in a rural population. Ann Trop Med Parasitol. 2008;102(7):585–95. 18817599 10.1179/136485908X355247

[21] Robertson LJ Hermansen L Gjerde BK Application of genotyping during an extensive outbreak of waterborne giardiasis in Bergen, Norway, during autumn and winter 2004. Appl Environ Microbiol. 2006;72(3):2212–7. 16517674 10.1128/AEM.72.3.2212-2217.2006 PMC1393178

[22] Matsubayashi M Kimata I Abe N Identification of genotypes of *Giardia intestinalis* isolates from a human and calf in Japan. J Vet Med Sci. 2005;67(3):337–40. 15805742 10.1292/jvms.67.337

[23] Abe N Nakamura S Kimata I An imported case of mixed infection by *Giardia* and *Cryptosporidium* parasites in Japan. Seikatsu Eisei. 2005;49(1):48–51.

[24] Ey PL Mansouri M Kulda J Genetic analysis of *Giardia* from hoofed Farm animals reveals artiodactyl-specific and potentially zoonotic genotypes. J Eukaryot Microbiol. 1997;44(6):626–35. 9435134 10.1111/j.1550-7408.1997.tb05970.x

[25] Cacciò SM Ryan U Molecular epidemiology of giardiasis. Mol Biochem Parasitol. 2008;160(2):75–80. 18501440 10.1016/j.molbiopara.2008.04.006

[26] Laishram S Kang G Ajjampur SS *Giardiasis:* A review on assemblage distribution and epidemiology in India. Indian J Gastroenterol. 2012;31(1):3–12. 22311296 10.1007/s12664-012-0161-9

[27] Volotão AC Costa-Macedo LM Haddad FS Genotyping of *Giardia lamblia* from human and animal samples from Brazil using (beta)-giardin gene: A phylogenetic analysis. Acta Trop. 2007;102(1):10–9. 17428432 10.1016/j.actatropica.2007.02.010

[28] Ravid Z Duque S Arévalo A Genetic diversity of *Giardia intestinalis* populations in Colombia. Biomedica. 2007;27(1):34–41. 17546222

[29] Bonhomme J Le Goff L Lemée V Limitations of tpi and bg genes sub-genotyping for characterization of human *Giardia lamblia* isolates. Parasitol Int. 2011; 60(3):327–30. 21627998 10.1016/j.parint.2011.05.004

[30] Cacciò SM De Giacomo M Pozio E Sequence analysis of the beta-giardin gene and development of a polymerase chain reaction-restriction fragment length polymorphism assay to genotype *Giardia lamblia* cysts from human faecal samples. Int J Parasitol. 2002;32(8):1023–30. 12076631 10.1016/s0020-7519(02)00068-1

[31] Eligio-García L Cortés-Campos A Jiménez-Cardoso E Classification of *Giardia intestinalis* isolates by multiple polymerase chain reaction (multiplex). Parasitol Res. 2008;103(4):797–800. 18551318 10.1007/s00436-008-1042-0

[32] Ponce-Macotela M Martínez-Gordillo MN Bermúdez-Cruz RM Unusual prevalence of the *Giardia intestinalis* A-II subtype amongst isolates from humans and domestic animals in Mexico. Int J Parasitol. 2002;32(9):1201–2. 12117503 10.1016/s0020-7519(02)00086-3

[33] Torres-Romero JC Euan-Canto Ade J Benito-González N Intestinal parasites and genotyping of *Giardia lamblia* in children: first report of genotype B in isolates from human clinical samples in Mexico. Mem Inst Oswaldo Cruz. 2014;109(3):388–90. 24676655 10.1590/0074-0276140507 PMC4131797

[34] Winkworth CL Learmonth JJ Matthaei CD Molecular characterization of *Giardia* isolates from calves and humans in a region in which dairy farming has recently intensified. Appl Environ Microbiol. 2008;74(16):5100–5. 18567681 10.1128/AEM.00232-08 PMC2519288

[35] Traub RJ Inpankaew T Reid SA Transmission cycles of *Giardia lamblia* in dogs and humans in Temple communities in Bangkok--a critical evaluation of its prevalence using three diagnostic tests in the field in the absence of a gold standard. Acta Trop. 2009;111(2):125–32. 19524080 10.1016/j.actatropica.2009.03.006

[36] Van Keulen H Olson B An overview of *Giardia* taxonomy: A historical perspective. *Giardia*: The Cosmopolitan Parasite. 2002; 283.

[37] Alyousefi NA Mahdy MA Xiao L Molecular characterization of *Giardia lamblia* in Yemen. Exp Parasitol. 2013;134(2):141–7. 23523861 10.1016/j.exppara.2013.03.001

[38] Minvielle MC Molina NB Polverino D First genotyping of *Giardia lamblia* from human and animal feces in Argentina, South America. Mem Inst Oswaldo Cruz. 2008;103(1):98–103. 18368240 10.1590/s0074-02762008000100015

[39] Yang R Lee J Ng J High prevalence *Giardia lamblia* assemblage B and potentially zoonotic subtypes in sporadic human cases in Western Australia. Int J Parasitol. 2010;40(3):293–97. 19703458 10.1016/j.ijpara.2009.08.003

[40] Asher AJ Holt DC Andrews RM Distribution of *Giardia lamblia* assemblages A and B among children living in a remote indigenous community of the Northern Territory, Australia. PLoS One. 2014:20;9(11):e112058. 25412502 10.1371/journal.pone.0112058 PMC4239041

[41] Haque R Roy S Kabir M *Giardia* assemblage A infection and diarrhea in Bangladesh. J Infect Dis. 2005;192(12):2171–3. 16288384 10.1086/498169

[42] Geurden T Levecke B Cacció SM Multilocus genotyping of *Cryptosporidium* and *Giardia* in non-outbreak related cases of diarrhea in human patients in Belgium. Parasitology. 2009;136(10):1161–8. 19631012 10.1017/S0031182009990436

[43] Ramírez JD Heredia RD Hernández C Molecular diagnosis and genotype analysis of *Giardia lamblia* in asymptomatic children from a rural area in central Colombia. Infect Genet Evol. 2015;32:208–13. 25795384 10.1016/j.meegid.2015.03.015

[44] Soliman RH Fuentes I Rubio JM Identification of a novel Assemblage B subgenotype and a zoonotic Assemblage C in human isolates of *Giardia intestinalis* in Egypt. Parasitol Int. 2011;60(4):507–11. 21989040 10.1016/j.parint.2011.09.006

[45] Foronda P Bargues MD Abreu-Acosta N Identification of genotypes of *Giardia intestinalis* of human isolates in Egypt. Parasitol Res. 2008;103(5):1177–81. 18622625 10.1007/s00436-008-1113-2

[46] Mukherjee A.K Karmakar S Raj D Multi-locus Genotyping Reveals High Occurrence of Mixed Assemblages in *Giardia lamblia* within a Limited Geographical Boundary. Brit Microbiol Res J. 2013; 3:190–97.

[47] Mohammed Mahdy A Surin J Mohd-Adnan A Molecular characterization of *Giardia lamblia* isolated from Semai Pahang Orang Asli (Peninsular Malaysia aborigines). Parasitology. 2009; 136(11):1237–41. 19660153 10.1017/S0031182009990527

[48] El Fatni C Olmo F El Fatni H First genotyping of *Giardia lamblia* and prevalence of enteroparasites in children from Tetouan (Morocco). Parasite. 2014;21:48. 25259605 10.1051/parasite/2014049 PMC4176428

[49] Lebbad M Ankarklev J Tellez A Dominance of *Giardia* assemblage B in Leon, Nicaragua. Acta Trop. 2008;106(1):44–53. 18325480 10.1016/j.actatropica.2008.01.004

[50] Robertson LJ Forberg T Hermansen L *Giardia lamblia* cysts isolated from wild moose and reindeer in Norway: genetic characterization by PCR-rflp and sequence analysis at two genes. J Wildl Dis. 2007;43(4):576–85. 17984252 10.7589/0090-3558-43.4.576

[51] Yason JA Rivera WL Genotyping of *Giardia lamblia* isolates among residents of slum area in Manila, Philippines. Parasitol Res. 2007; 101(3):681–7. 17401723 10.1007/s00436-007-0533-8

[52] Boontanom P Mungthin M Tan-Ariya P Epidemiology of giardiasis and genotypic characterization of *Giardia lamblia* in preschool children of a rural community, central Thailand. Trop Biomed. 2011;28(1):32–9. 21602766

[53] Breathnach AS McHugh TD Butcher PD Prevalence and clinical correlations of genetic subtypes of *Giardia lamblia* in an urban setting. Epidemiol Infect. 2010;138(10):1459–67. 20144251 10.1017/S0950268810000208

[54] Lebbad M Petersson I Karlsson L Multilocus genotyping of human *Giardia* isolates suggests limited zoonotic transmission and association between assemblage B and flatulence in children. PLoS Negl Trop Dis. 2011;5(8):e1262. 21829745 10.1371/journal.pntd.0001262 PMC3149019

[55] Bertrand I Albertini L Schwartzbrod J Comparison of two target genes for detection and genotyping of *Giardia lamblia* in human feces by PCR and PCR-restriction fragment length polymorphism. J Clin Microbiol. 2005; 43(12):5940–4. 16333079 10.1128/JCM.43.12.5940-5944.2005 PMC1317193

[56] Cooper MA Sterling CR Gilman RH Molecular analysis of household transmission of *Giardia lamblia* in a region of high endemicity in Peru. J Infect Dis. 2010;202(11):1713–21. 20977340 10.1086/657142 PMC2974043

